# Automated image computing reshapes computational neuroscience

**DOI:** 10.1186/1471-2105-14-293

**Published:** 2013-10-04

**Authors:** Hanchuan Peng, Badrinath Roysam, Giorgio A Ascoli

**Affiliations:** 1 Allen Institute for Brain Science, Seattle, WA, USA; 2 Department of Electrical and Computer Engineering, University of Houston, Houston, TX, USA; 3 Krasnow Institute for Advanced Study, George Mason University, Fairfax, VA, USA

## Abstract

We briefly identify several critical issues in current computational neuroscience, and present our opinions on potential solutions based on bioimage informatics, especially automated image computing.

## 

Computational neuroscience is undergoing a transformation. Traditionally, this field has focused on studying information processing in nervous systems by collecting, analyzing, and simulating neuronal electrophysiology data [1,2]. Increasingly, the emphasis is shifting towards neuromorphological pattern analysis and brain atlas modeling using advanced microscopy and image computing. One important motivation for this changing focus is the need to ground our understating of animal behavior in real three-dimensional (3D) neuronal morphologies and connectivity. When dynamic imaging of living nervous tissue is possible, there is an additional opportunity to investigate the time-dependent molecular mechanisms in brain tissue in a data-driven manner. Advances in automated image computing are making such studies practical and informative.

## Image computing is critical for mapping brain and building neuron databases

A common goal among many current studies is to map brain anatomy by systematically characterizing the distribution, projection, and connectivity of neurons throughout a nervous system. Pursuing this ambitious goal is increasingly practical, due to converging advances in neuron labeling [3,4], histological preparation [5], multi-dimensional imaging [6,7], and image computing [8,9]. Several large-scale 3D imaging-based computational neuroscience initiatives, such as the HHMI Janelia FlyLight (http://janelia.org/team-project/fly-light) and FlyEM projects (http://janelia.org/team-project/fly-em), the Allen Mouse Brain “MindScope” [10] and Connectivity Atlas (http://alleninstitute.org/science/public_resources/atlases/connectivity_atlas.html), and the Harvard mouse brain connectome project (http://cbs.fas.harvard.edu/science/connectome-project), have been launched to acquire massive datasets, in which each image contains hundreds of millions of pixels.

The task of storing, organizing, and disseminating these vast datasets has driven the development of several neuronal databases and digital brain atlases. For example, http://NeuroMorpho.Org is a web-accessible database that allows researchers to share neuron reconstructions, quantify neuronal morphology in a standardized manner, mine the resulting morphological features, and use them in computational modeling and neuronal network simulation studies [11]. The digital representations of light-level single neurons in http://NeuroMorpho.Org is associated with rich metadata, but only coarse information about the cellular location and orientation relative to surrounding brain structures. In contrast, 3D digital brain atlases contain rich and integrated information on the spatial coordinates of 3D reconstructed neurons, gene expression, functional modules, and networks, all mapped into a ‘standard’ view of brain anatomy. Examples of recent digital atlases include a *C. elegans* connectome [12] produced from electron microscopic images, a large-scale single-neuron atlas (http://flycircuit.org[13]) and a stereotypy atlas of major neurite tracts [14] of the adult fruit fly brain, both produced from confocal microscopic images, a genome-wide gene expression map of the mouse brain produced using wide-field microscopic images (http://brain-map.org[15]), and a high-resolution reconstruction of the rat hippocampus [16].

**Figure 1:**
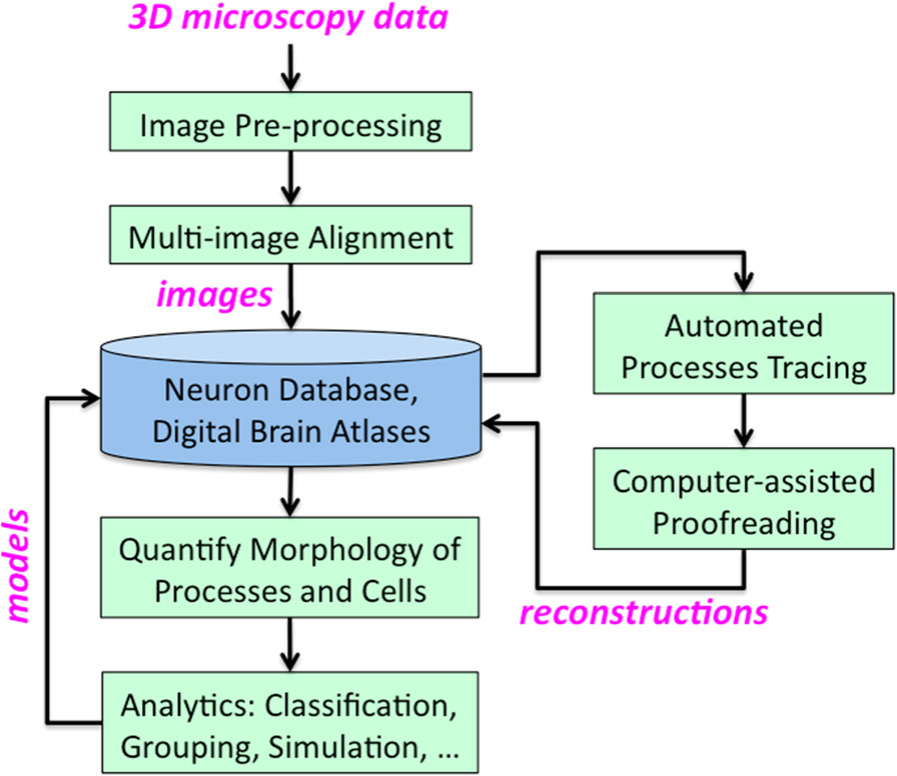
**Major steps in building digital neuronal databases and brain atlases.** After necessary image processing, the 3D morphology of a neuronal pattern is reconstructed from microscopy data. Multiple neuronal patterns need to be organized based on brain regions and neuronal types. For brain atlases, these reconstructions are mapped into a common 3D spatial coordinate system. This may involve an iterative analysis process.

Producing databases and atlases of neuronal structures from microscopy data entails a pipeline of image processing and analysis (Figure 1) (also see [9]). Key steps include (1) image pre-processing: correct images for imaging artifacts and standardize the raw data; (2) registration: align brain morphology and stitch together separately imaged brain regions; (3) segmentation and validation: trace neuron morphology and ensure that the reconstructions are correct; (4) feature extraction: make detailed quantitative measurements of traced neurons; and (5) analytics: model, infer, and predict the identities of neurons or neuronal patterns using morphology, location, genetic, lineage, and other extracted features. Many of these steps, such as 3D neuron tracing, are also widely used in smaller-scale analysis of neuronal phenotypes.

## The challenge of full automation

The long-standing need to automate the laborious and subjective manual analysis of light-microscopic and electron-microscopic images has motivated a large number of bioimage informatics efforts [8,17-21]. The recent spurt in imaging throughput, combined with the desire for large-scale computational modeling, has added a sense of urgency to this need. For instance, manual tracing of neuron morphology is prohibitively expensive for analyzing image data that is approaching the scale of terabytes and thousands of image stacks, let alone mining the higher-order associations in these data.

For computational neuroscience, this great demand motivated several major institutions to conduct the worldwide DIADEM Challenge (http://diademchallenge.org) in 2010 as a way to stimulate progress and attract new computational researchers to join the technology development community. The challenge was to develop algorithms capable of automatically tracing stacks of images visualizing the tree-like shape of neuronal axons and dendrites into 3D digital reconstructions. This competition succeeded in stimulating a burst of progress. Five finalist-teams were selected from more than one hundred entries. A novel metric methodology was custom designed to compare automated and manual reconstructions [22]. While none of the algorithms presented at the finishing stage reached the official goal of a 20-fold speed-up in the reconstruction process, all finalists’ software programs were made publicly downloadable along with the challenge data sets and the DIADEM metric, providing a useful toolbox for continuous and future development.

To achieve full automation, libraries of computer programs for neuronal pattern extraction, comparison and inference must be integrated into toolkits for multi-dimensional image visualization and analysis (e.g. http://vaa3d.org[23]; http://farsight-toolkit.org). Optimizing each step of automated image computing (Figure 1) is also important to ensure a lower error rate compared to humans. In addition to a metric of precision, automation of image computing should also be assessed using measures of the speed-up compared to manual work, as well as the robustness of the method with respect to changes of its parameters and the type and level of noise in the data. These aspects relate to the scalability to large-scale applications and the generalization to similar or new problems. The above considerations apply to image data produced by most imaging modalities. Despite considerable progress, many computational problems remain open. For instance, the DIADEM neuron reconstruction challenge stands unmet. Part of the reason could be that only relatively small image data sets on neuron morphology were previously available to researchers. We hope this situation will change soon due to the availability of benchmark datasets and the strong demand of very large-scale single neuron screening projects in the next five years.

## Making computer programs more intelligent

Increasingly, researchers have developed ways to make some of these algorithms more intelligent. Domain-specific prior knowledge can be used to improve the “intelligence” of an algorithm, akin to the observation that a “supervised” machine-learning algorithm generally performs better (e.g. predicts more accurately) than an “unsupervised” one. How to incorporate domain knowledge in an algorithm? One way is to code this information into explicit rules. For instance, the spatial location of cells coded as directed graphs has been used as constraints in training a nuclear image segmentation and recognition algorithm [24]. In another example, particular types of neuron tracing errors could be propagated through a large set of results to identify other potential erroneous reconstruction loci [25]. However, in many cases it is hard to enumerate all possible rules and code them explicitly. Rules may also become contradictory, biased, or “fuzzy” under different circumstances. Therefore, another more competent way to provide the domain knowledge is needed.

Interesting solutions may originate from comprehensive neuron databases and brain anatomy atlases. The reciprocal relationship that links neuroscience databases and atlases with image computing methods offers a synergistic opportunity to improve both of them iteratively. In both atlases and databases, it is possible to quantify neuronal patterns from many samples statistically. The statistics provide good “rules” or “priors”, and new paradigms, for improving image-computing algorithms. For example, since an atlas of cells is a comprehensive model of the expected yet spatially deformed objects (cells) in the observed image data, a model-based search approach can be designed to achieve registration, segmentation, and recognition at the same time. An automated algorithm for simultaneous cell segmentation and recognition of *C. elegans* cells [26] uses a standard 3D nuclear atlas of this animal to achieve much better performance than the more intuitive approach of performing these tasks in separate steps.

When neuron locations are stereotypical, atlas-based image-computing methods can be powerful to design novel biological experiments. The spatial variation of most nuclei, including many neurons, in freshly hatched *C. elegans* is about 2 microns [24], just about the average size of a nucleus. For adult fruit fly brains, this variation for the major neurite tracts is merely ~3 microns, orders of magnitude smaller than the dimensions of an entire brain [14]. Therefore, it is possible to design new computer-driven instruments to repeatedly target the same neuron or neurite tract, locally perturb the neuronal circuits of these animals in real time experiments, and observe the response of the nervous system. Among ongoing efforts, the Janelia SmartScope project aims at leveraging new image-guided computer programs to drive a 3D laser-scanning-microscope to target and stimulate single cells precisely in 3D and observe their downstream activities, using optogenetics and calcium imaging.

## Studying dynamics and functions of neural circuits

Functional connectivity and dynamics of neuronal connections are two other important topics of computational neuroscience. Neuronal dynamics are reflected in structure and activity at a variety of temporal scales, ranging from arbor growth and cell migration (days to years), through dendritic spine twitching and axonal bouton crawling (minutes to hours), to neuron firing (milliseconds to seconds). Intravital imaging [27] provides excellent means to visualize these temporal changes. The dynamics can then be studied using four-dimensional computational image analysis. For instance, tracking the 3D morphological change of neurons over time from time-lapse imaging data offers great opportunity to improve neuronal classification and characterize the structural plasticity of growing, degenerating, or regenerating neurons [20]. Anchoring corresponding branching points of varying neuron morphology and detecting persistent and variable structures (branch tips, boutons, spines) from multi-dimensional image series are useful steps to analyze neuronal dynamics. Although the automation of these image analysis tasks is still new, it is highly desired to quantitatively study these problems at large scale.

In another example, 3D segmentation and tracking of somatic calcium imaging data provide a powerful way to study neuron activity patterns. Computational causal analysis might also enable interesting analysis of synaptic connections [28] (see however [29]). Imaging neuronal firing with possibly genetically encoded voltage-gated dyes or fast calcium indicators could enable the co-detection of neural network structure and activity. Using brain atlases and computational image-guided analysis, it is possible to pinpoint the firing sites *in vivo* and record the firing patterns in real-time experiments. This approach could complement the recent non-image-guided automatic whole-cell patch-clamp electrophysiology [30].

Correlative analysis of microscopic image data of neurons and other functional data, such as electrophysiology and optical-physiology recording and behavioral assays, can become a powerful approach to gain useful information and knowledge from a large amount of data, and may lead to the ultimate understanding of how individual neurons and their networks work. For instance, digitization of the dragonfly descending neurons’ morphology from confocal images has been correlated with the electrical recording and measurement of the receptive fields of these neurons [31]. This analysis led to a new finding that the average population vector of the neuronal response of a surprisingly small number of descending neurons actually precisely controls the wings of the animal in targeting preys [31]. On the other hand, for behavior assays, systematic video-analysis based high-throughput studies have yielded powerful new ways to associate the neuronal circuits of both insects and mammals with either the stimuli (such as the mouse whisker system [32]) or the behavior output (such as the *Drosophila* trajectories [33]). In the work of [33], a machine learning system enables biologists to automatically annotate the behaviors of animals in each frame of a video. It can be used to detect a wide variety of social and locomotion behaviors across flies, mice, and larvae.

Recent years have witnessed an acceleration of the ongoing transformation of computational neuroscience due to image computing automation. For example, neurite outgrowth assays employing automated reconstruction of neurons in high-throughput high-content environments are now common, and are used for testing hypotheses, screening, and toxicology studies [34-36]. At a more advanced level, new high-throughput methods for accurate neurite reconstruction [37] have allowed analysis of the specific wiring underlying computation of direction-selectivity in retinal circuits [38]. More generally, these kinds of structural neurobiology studies may systematically provide the much needed mechanistic understanding of neural computation [39] if employed in large-scale automated applications [40] of new techniques for the anatomical and molecular interrogation of intact biological systems [41].

## Future prospects

We are optimistic that in the next five years more automated and intelligent computer programs for analyzing large-scale brain images will yield tangible advancements in computational neuroscience. The neuron anatomy projects for various model animals will start to deliver integrated data sets, organized in neuron databases and brain atlases. This will provide invaluable data resources for the research community to analyze. This may lead to a paradigm shift similar to the massive rise of mining and reanalysis of ‘secondary’ (archived) data following the sudden abundance of genome sequences 10 years ago. Neuron databases and brain atlases are also going to incorporate more dynamic and functional information, such as from time-lapse and calcium imaging of interacting neurons. More realistic computational models of neurons and their networks may foster a deeper understanding of the functioning mechanisms of nervous systems, paving the path to re-create a brain in silico.
